# Pan-genome analysis of three main Chinese chestnut varieties

**DOI:** 10.3389/fpls.2022.916550

**Published:** 2022-07-25

**Authors:** Guanglong Hu, Lili Cheng, Yunhe Cheng, Weitao Mao, Yanjie Qiao, Yanping Lan

**Affiliations:** Engineering & Technology Research Center for Chestnut of National Forestry and Grassland Administration, Key Laboratory of Biology and Genetic Improvement of Horticultural Crops (North China) of Ministry of Agriculture, Beijing Engineering Research Center for Deciduous Fruit Trees, Institute of Forestry and Pomology, Beijing Academy of Agriculture and Forestry Sciences, Beijing, China

**Keywords:** *Castanea mollissima*, *de novo* assembly, pan-genome, waxy gene, Nanopore sequencing, genome database

## Abstract

Chinese chestnut (*Castanea mollissima* Blume) is one of the earliest domesticated plants of high nutritional and ecological value, yet mechanisms of *C. mollissima* underlying its growth and development are poorly understood. Although individual chestnut species differ greatly, the molecular basis of the formation of their characteristic traits remains unknown. Though the draft genomes of chestnut have been previously released, the pan-genome of different variety needs to be studied. We report the genome sequence of three cultivated varieties of chestnut herein, namely Hei-Shan-Zhai-7 (H7, drought-resistant variety), Yan-Hong (YH, easy-pruning variety), and Yan-Shan-Zao-Sheng (ZS, early-maturing variety), to expedite convenience and efficiency in its genetics-based breeding. We obtained three chromosome-level chestnut genome assemblies through a combination of Oxford Nanopore technology, Illumina HiSeq X, and Hi-C mapping. The final genome assemblies are 671.99 Mb (YH), 790.99 Mb (ZS), and 678.90 Mb (H7), across 12 chromosomes, with scaffold N50 sizes of 50.50 Mb (YH), 65.05 Mb (ZS), and 52.16 Mb (H7). Through the identification of homologous genes and the cluster analysis of gene families, we found that H7, YH and ZS had 159, 131, and 91 unique gene families, respectively, and there were 13,248 single-copy direct homologous genes in the three chestnut varieties. For the convenience of research, the chestnut genome database^1^ was constructed. Based on the results of gene family identification, the presence/absence variations (PAVs) information of the three sample genes was calculated, and a total of 2,364, 2,232, and 1,475 unique genes were identified in H7, YH and ZS, respectively. Our results suggest that the *GBSS II-b* gene family underwent expansion in chestnut (relative to nearest source species). Overall, we developed high-quality and well-annotated genome sequences of three *C. mollissima* varieties, which will facilitate clarifying the molecular mechanisms underlying important traits, and shortening the breeding process.

## Introduction

In 2020, at least 720 million people (≥ 9.9% of the global population) faced hunger, this represents an increase over previous years, and the greatest percentage of the total population since 2010 (FAO, IFAD, UNICEF, WFP, and WHO, 2021). Because of the ongoing climate change as well as the increasing global population and the COVID19 pandemic, the number of people facing hunger is expected to rise significantly. To alleviate global hunger, more attention needs to be given to non-staple food crops (Chapman et al., 2022). Historically, chestnuts was promoted to fight hunger (Gabriele et al., 2020). The XVIIIth century is considered by many as the worst century of hunger, because of which the chestnut tree tirelessly renewed its aid and continued to feed mountain residents (Adua, 1999).

Chinese chestnut (*C. mollissima* Blume; Fagaceae) has been cultivated for more than 6,000 years in the Banpo Ruins of Xi’an, China, according to archeological findings (Hao and Zhang, 2014). Chestnut is an important tree species currently cultivated in eastern Asia, both for its ecological and economic advantages. China is considered a gene center for the genus *Castanea* (Vavilov, 1952; Zhang et al., 2015). The chestnut is a traditional nut and also a popular food around the world (Guo et al., 2019). China is one of the top producers of chestnuts (Wang et al., 2020b). Over 300 cultivars have been selected for nut production (Li et al., 2009) Many characteristics of the chestnut plant affect its growth and development which in turn affects the development of the chestnut industry.

Presently, most of the chestnut varieties sold in the market are mid- and late-maturing, which cannot adequately meet the diversified needs of the market. Early-maturing chestnut varieties could be put on the market earlier, which would greatly improve the overall value of the nut (Cao, 2015) However, only a few early maturing cultivars are available in the market, which have the disadvantages of not being drought tolerant and not easily pruned. Breeding Early-maturing cultivars that are drought-resistant and easy-pruning is a priority for chestnut breeding (Ren and Jia, 2014; Zhao and Zhang, 2015). Fortunately we have bred three main cultivation varieties namely Hei-Shan-Zhai-7 (drought-resistant variety; Huang et al., 2009), Yan-Hong (easy-pruning variety; Gao et al., 1980), and Yan-Shan-Zao-Sheng (early-maturing variety; Cheng et al., 2013). If more varieties with early maturity, drought resistance and easy-pruning characteristics are sequenced, it will expedite clarifying the molecular mechanisms underlying these traits and shortening the breeding process.

Starch is one of the most important components of a chestnut, and accounts for 50–80% of its dry matter content (Liu et al., 2015). Chestnut starch is considered as a potentially functional component of dietary fiber, which may be sources of resistant starch, thus improving health (Liu et al., 2022). Given the rapid development of starch-based foods, chestnut starch shows increasing application potential. There have been numerous studies on chestnut as a new source of starch (Liu et al., 2015, 2019). The characteristics of chestnut starch vary greatly with the variety and its geographical distribution (Long et al., 2018). Waxiness is one of the most important edible qualities of chestnuts; however, this trait also varies greatly with the genotype and production area. The proportion of amylopectin and amylose in chestnut kernel starch varies among cultivars (Liang, 2011). However, there are few reports on waxy genes in chestnut due to the lack of genome sequence information.

There has been a rapid increase in the number of pan-genome studies on plants. The first published plant pan-genome was based on a comparison of whole-genome assemblies of seven wild soybean (*Glycine soja*) accessions (Li et al., 2014). Simultaneously, another study examined the pan-genome of rice (*Oryza sativa*), based on three divergent accessions (Schatz et al., 2014). In recent years, there has been a surge in plant genome sequencing projects and in the comparison of multiple related individuals. The high degree of genomic variation observed among individuals belonging to the same species led to the realization that single reference genomes do not represent the diversity within a species, which in turn led to the expansion of the pan-genome concept. Pan-genomes represent the genomic diversity of a species, and include core genes (i.e., genes found in all individuals) as well as variable genes (i.e., genes absent in some individuals). Genes involved in biotic and abiotic stress responses are commonly enriched within the variable gene groups. The growth of pan-genomics in plants and exploration of gene presence/absence variations (PAVs) can support plant breeding and evolutionary studies (Bayer et al., 2020).

Although the genome sequence of Chinese chestnut has been reported previously (LaBonte et al., 2018; Xing et al., 2019; Sun et al., 2020; Wang et al., 2020a), higher quality genome assembly and pan-genome analysis are required. In the present study, we generated high-quality chromosome-level reference genome assemblies of three *C. mollissima* varieties, namely Hei-Shan-Zhai-7 (drought-resistant variety), Yan-Hong (easy-pruning variety), and Yan-Shan-Zao-Sheng (early-maturing variety), using Oxford Nanopore Technology (ONT) and Illumina HiSeq X sequencing and Hi-C mapping, subsequently, we performed a pan-genome analysis and constructed a chestnut genome database. These results will help reveal differences in the traits of the three varieties and will support breeding programs aimed at the genetic improvement of chestnuts.

## Materials and methods

### Sampling collection and sequencing

Three chestnut including Hei-Shan-Zhai-7 (H7), Yan-Hong (YH), and Yan-Shan-Zao-Sheng (ZS) were used in this study. Healthy leaves were collected from the tress of all three varieties growing in Shachang Village (40.3875°N, 117.0275°E), Miyun District, Beijing, China. The freshly harvested samples were immediately frozen in liquid nitrogen. High-quality and high-molecular-weight genomic DNA was extracted from the frozen leaves using the cetyltrimethylammonium bromide (CTAB) method (Yan et al., 2018). The quality and concentration of the extracted genomic DNA were examined by 1% agarose gel electrophoresis and with a Qubit fluorimeter (Invitrogen, Carlsbad, CA, United States). This high-quality DNA was used for subsequent Nanopore and Illumina sequencing.

### Library construction and genome sequencing

Approximately 15 μg of genomic DNA was subjected to size selection using the BluePippin system (Sage Science, Beverly, MA, United States), and the size-selected 30–80-kb fragments were processed using the Ligation Sequencing Kit 1D (SQK-LSK109), according to the manufacturer’s instructions, to generate ONT long-reads. Briefly, DNA fragments were repaired using the NEBNext FFPE Repair Mix (New England Biolabs, Ipswich, MA, United States). After end reparation and 3′-adenylation with the NEBNext End Repair/dA-Tailing Module reagents, ONT sequencing adapters were ligated to the fragments using the NEBNext Quick Ligation Module (E6056). The final library was sequenced on three different R9.4 flow cells using the PromethION DNA sequencer (Oxford Nanopore, Oxford, United Kingdom) for 48 h. The MinKNOW software (version 2.0) was used to conduct base calling from the raw signal data and to convert the fast5 files into fastq files. The resultant raw data were then filtered to remove reads less than 5 kb in size (short reads) and those containing low-quality bases and adapter sequences.

### Illumina sequencing

Paired-end (PE) libraries, with 300-bp insert size, were constructed according to the Illumina standard protocol (San Diego, CA, United States), and subjected to PE (2 × 150 bp) sequencing on the Illumina HiSeq X Ten platform (Illumina, San Diego, CA, United States). Reads with low-quality bases, adapter sequences, and duplicated sequences were discarded, and the resultant clean reads were used for all subsequent analyses.

### Genome assembly

Canu (version 1.5; Koren et al., 2017) was used to perform the initial read correction, and genome assembly was constructed using Wtdbg.^2^ The consensus assembly was generated using two rounds of Racon (version 1.32; Robert et al., 2017) and three rounds of Pilon (version 1.21; Walker et al., 2017), which polished the Illumina reads using default settings.

### Hi-C library construction and sequencing

We constructed Hi-C fragment libraries as described previously. (Rao et al., 2014). Briefly, the leaf tissues were fixed in formaldehyde, and then treated with *Hin*dIII restriction endonuclease to digest all DNA. The 5′ overhang of each fragment was repaired, labeled with biotinylated nucleotides, and ligated in a small volume. After reversing the crosslinks, the ligated DNA was purified and sheared to a length of 300–700 bp. The DNA fragments exhibiting interaction were captured with streptavidin beads and prepared for Illumina sequencing. The final Hi-C libraries were sequenced on the Illumina HiSeq X Ten platform (Illumina, San Diego, CA, United States) to obtain 2 × 150 bp PE reads. The quality of the Hi-C data was assessed through a two-step process. First, an insert fragment frequency plot was constructed to detect the quality of the Illumina sequencing. Then, BWA-MEM (version 0.7.10-r789; Li and Durbin, 2009) was used to align the clean PE reads to the construct the genome assembly draft. Finally, Hi-C-Pro (version 2.10.0; Servant et al., 2015) was used to find all valid reads from unique mapped read pairs.

### Chromosomal-level genome assembly using Hi-C data

To correct contig error, a preassembly was generated by breaking the contigs into segments with an average length of 500 kb and then mapping the Hi-C data to these segments using BWA-MEM (version 0.7.10-r789; Li and Durbin, 2009). The corrected Hi-C contigs and valid reads were used to perform chromosomal-level genome assembly using LACHESIS (Burton et al., 2013) with the following parameters:

CLUSTER_MIN_RE_SITES = 22;CLUSTER_MAX_LINK_DENSITY = 2;CLUSTER_NONINFORMATIVE_RATIO = 2;ORDER_MIN_N_RES_IN_TRUNK = 10;ORDER_MIN_N_RES_IN_SHREDS = 10.

A genome-wide Hi-C heatmap was generated for each varieties using ggplot2 in the R package to evaluate the quality of the chromosomal-level genome assembly.

### Assessment of the genome assemblies

The Illumina reads were first aligned to the filefish assembly using BWA-MEM (version 0.7.10-r789; Li and Durbin, 2009) to assess genome assembly completeness and accuracy. Subsequently, CEGMA (version 2.5; Parra et al., 2007) was used to find core eukaryotic genes (CEGs) in the genome, with the identity parameter set to >70%. Finally, the completeness of the genome assembly was evaluated using benchmarking sets of universal single-copy orthologs (BUSCO; version 2.0; Simão et al., 2015).

### Repeat annotation, gene prediction, and gene annotation

Because of the relatively low conservation of interspecies repeat sequences, a specific repeat sequence database needs to be constructed to predict species-specific repeat sequences. LTR-FINDER (version 1.05; Xu and Wang, 2007) and RepeatScout (version 1.0.5; Bai, 2007) were used to identify repetitive sequences in the chestnut genome sequences assembled in this study. Then, a repeat sequence database was constructed based on the principles of structural and *de novo* repeat prediction. These predicted repeats were classified using PASTEClassifer (version 1.0; Claire et al., 2017), and then merged with the Repbase database (version 19.06; Jurka et al., 2005) to create the final repeat database. Finally, RepeatMasker (version 4.0.6; Tarailo Graovac and Chen, 2009) was used to detect all repetitive sequences in the chestnut genome from that database with the following parameters: “-nolow -no_is -norna -engine wublast.”

The genomic structure of the three Chinese chestnut varieties was determined using three approaches: *ab initio* prediction, homologous sequence search, and unigene predictions. The *ab initio* prediction was performed with Genscan (Burge and Karlin, 1997), Augustus (version 2.4; Stanke and Waack, 2003), GlimmerHMM (version 3.0.4; Majoros et al., 2004), GeneID (version 1.4; Blanco et al., 2007), and SNAP (version 2006-07-28; Korf, 2004). To predict genes in chestnut varieties based on homology, GeMoMa (version 1.3.1; Keilwagen et al., 2016; Jens et al., 2018) was used to search the genomes of *Arabidopsis thaliana*, *O. sativa*, *Quercus robur*, and *Fraxinus excelsior*. Then, based on these referenced transcripts, the chestnut genome assemblies were screened using Hisat (version 2.0.4; Kim et al., 2015), Stringtie (version 1.2.3; Pertea et al., 2015), TransDecoder (version 2.0),^3^ and GeneMarkS-T (version 5.1; Tang et al., 2015). PASA (version 2.0.2; Campbell et al., 2006) was used to predict unigene sequences, without reference assembly, based on transcriptome data. Finally, the results obtained using the above methods were integrated by EVM (version 1.1.1; Haas et al., 2008), and modified with PASA (version 2.0.2; Campbell et al., 2006).

The predicted gene sequences were then compared with non-redundant (NR) protein sequences at the National Center for Biotechnology Information (NCBI; Marchler et al., 2011), euKaryotic Orthologous Groups of proteins (KOG; Koonin et al., 2004), Gene Ontology (GO; Dimmer et al., 2012), Kyoto encyclopedia of genes and genomes (KEGG; Kanehisa and Goto, 2000), and TrEMBL (Boeckmann et al., 2003) functional databases using BLAST (version 2.2.31; Altschul et al., 1990) with an e-value cutoff of 1E−5. Non-coding RNA, microRNA, and ribosomal RNA (rRNA) sequences were predicted by genome-wide alignment using BLAST (version 2.2.31; Altschul et al., 1990) based on the Rfam database (version 1.3.1; Griffiths et al., 2005). Transfer RNAs (tRNAs) were identified using tRNAscan-SE (version 1.3.1; Lowe and Eddy, 1997).

### Comparative genomics

To resolve the phylogenetic position of the *C. mollissima* varieties (YH, H7, and ZS), OrthoMCL (version 2.0.9; Li et al., 2003) was first used to detect orthologous groups by retrieving the protein data of 10 plant species: Chinese chestnut (*C. mollissima*; Xing et al., 2019), summer squash (*Cucurbita pepo*; Montero et al., 2018), wild pear (*Pyrus betulifolia*; Dong et al., 2020), mulberry (*Morus notabilis*; He et al., 2013), peach (*Prunus persica*; Verde et al., 2013), oak (*Q. robur*; Plomion et al., 2018), indica rice (*O. sativa* subsp. *indica*; Du et al., 2017), mei (*Prunus mume*; Zhang et al., 2012), horsetail she-oak (*Casuarina equisetifolia*; Ye et al., 2019), and apple (*Malus domestica*; Zhang et al., 2019). Then using the single-copy protein sequences of *C. mollissima* (H7, YH, and ZS) and nine other chestnut species, an evolutionary tree was constructed using PHYML (version 3.0; Stéphane et al., 2010). The divergence time among species was estimated using the MCMCTree program of the PAML (version 4.0) package (Yang, 2007), and gene families that underwent expansion or contraction were identified using CAFÉ (version 4.0; de Bie et al., 2006). Collinearity analysis with the genome of *Q. robur* (parameter: -l 10,000, other parameters are default), and visualization of differences in size among the three genomes, the MUMmer software (Kurtz et al., 2004) was used to identify similar regions.

### Pan-genome of three varieties of Chinese chestnut

Pan-genome enables the exploration of genetic variation and diversity among species, which is essential to fully understand the genetic control of phenotypes (Lu et al., 2015). Blastp (version 2.7.1; Jacob et al., 2008) was used to compare all protein sequences of the three chestnuts, with the following parameter: “-evalue 1e-5.” Then, OrthoMCL (version 2.0.9) was used to identify homologous genes according to the comparison results. Finally, OrthoMCL (McL-14-137) was used to cluster the gene families, with the following parameters: “-I 1.5” and “-TE 20.”

### Construction of the chestnut genome database

The Chestnut Genome Database was set up using Tomcat and MySQL. The backend was designed and implemented using the SpringBoot + MyBatis framework, with CentOS as the server. Data were visualized using an open source ECharts package. The genomic data of four chestnut varieties, H7, YH, ZS, and N11-1(Wang et al., 2020a), have been included in this database.

### Characterization of waxy genes (*GBSS II*) in *Castanea mollissima*

The reference genome sequences and gene structure annotation information of *C. mollissima* varieties were downloaded from the Chestnut Genome Database (See Footnote 1). All protein sequences encoded by the waxy gene family were downloaded from the SwissProt database. With-evaluate is set to 1e-5, then blastp is used to search all possible waxy homology in *C. mollissima* (Altschul et al., 1990). We have also employed the HMMER web server (Finn et al., 2011). All public available waxy protein sequences were aligned using the MUSCLE software (Edgar, 2004) with default parameters. The Hidden Markov Model (HMM) model was constructed with the alignment results. Waxy genes sequences identified using BLAST and HMM method were then combined for further motif and domain analyses. The MEME software (Timothy et al., 2015) was employed to identify conserved motifs. Phylogenetic trees were constructed using IQtree (Lam-Tung et al., 2015). Conserved domains were predicted on the NCBI CDD database (Marchler-Bauer et al., 2015); All abovementioned results were visualized using TBtools software (Chen et al., 2020). With the help of TBtools, we have found two waxy genes were mis-assembled as one. Gene structure prediction and curation were conducted using the Fgenesh (Solovyev et al., 2006) software. With the high-quality waxy gene structure annotation, the gene position, exon number, and open reading frame (ORF) length were summarized using the GXF Stat function of the TBtools software. The subcellular localization of the GBSS protein family members was predicted using the CellO (Yu et al., 2006) software.

### Data availability statement

The sequencing datasets and genome assemblies have been deposited in public repositories. The Illumina genome sequencing data were deposited in the NCBI Sequence Read Archive under the accession numbers SRR16288271 (Hei-Shan-Zhai-7), SRR16288268 (Yan-Hong) and SRR16288265 (Yan-Shan-Zao-Sheng). The Nanopore genome sequencing data were deposited in the NCBI Sequence Read Archive under the accession numbers SRR16288270 (Hei-Shan-Zhai-7), SRR16288267 (Yan-Hong) and SRR16288264 (Yan-Shan-Zao-Sheng). The Hi-C sequencing data were deposited in the NCBI Sequence Read Archive under the accession numbers SRR16288269 (Hei-Shan-Zhai-7), SRR16288266 (Yan-Hong) and SRR16288263 (Yan-Shan-Zao-Sheng). The URL links of accession numbers are listed in Supplementary Table S17.

## Results and discussion

### Genome assembly

Based on the distribution of 21-mers among the Illumina HiSeq reads. The genomes of *C. mollissima* were estimated to be 664.89 Mb (YH), 628.90 Mb (H7) and752.70 Mb (ZS), with approximately 0.98% (YH), 1.05% (H7) and 0.60% (ZS) heterozygosity. The k-mer distribution curve peaked at a depth of 57 (zs), 51 (YH) and 58 (H7), with a k-mer number of 34,316,017,419(YH), 36,619,119,572 (H7) and 43,087,876,811 (ZS; Supplementary Figure S1).

Three varieties of Chinese chestnut (YH, H7, and ZS) were sequenced using PromethION DNA sequencer. Overall, approximately 95.01, 99.22, and 83.62 Gb of clean data at a total sequencing depth of approximately 104×, 126×, and 122× were obtained for YH, H7, and ZS, respectively.

Nanopore’s third-generation data were corrected to obtain high-accuracy data. Canu (version 1.5; Koren et al., 2017) was used to perform the initial read correction, and genome assembly was constructed using Wtdbg. The consensus assembly was generated using two rounds of Racon (version 1.32; Robert et al., 2017) and three rounds of Pilon (version 1.21; Walker et al., 2017), which polished the Illumina reads using default settings. The total lengths of the genome sequences were determined to be 679.87 Mb with a contig N50 of 3.65 Mb (YH), 790.99 Mb with a contig N50 of 2.17 Mb (ZS), and 687.24 Mb with a contig N50 of 3.39 Mb (H7; Table 1).

**Table 1:** Summary of three *C. mollissima* genomes assembly.

Parameter	YH	H7	ZS
No. of contigs	1,514	1,460	827
Contig length (bp)	679,866,993	687,236,598	790,986,026
N50 (bp)	3,649,215	3,389,933	2,174,699
N90 (bp)	330,218	448,929	436,758
Contig max (bp)	24,666,180	21,536,999	14,385,919

Hi-C libraries were sequenced on the Illumina sequencing platform using the Sequencing By Synthesis (SBS) technology, generating 325,605,014 (YH), 295,593,125 (ZS), and 284,973,447 (H7) reads.

To evaluate the quality of the Hi-C data, we plotted the frequencies of insert fragment length (Supplementary Figure S2). The fragment length distribution curve of all three varieties showed a peak at approximately 300 bp, which is consistent with the target size, and the peak type was narrow. Approximately 84.24% (YH), 90.36% (ZS), and 89.98% (H7) of the Hi-C read pairs could be successfully mapped on to the genome, and 62.01% (YH), 59.63% (ZS), and 56.15% (H7) of the read pairs could be uniquely mapped.

Our analyses showed 201,899,176 (YH), 176,262,008 (ZS), and 160,005,850 (H7) read pairs were uniquely correlated with the genome, respectively. Among these, 104,212,288 (YH), 129,536,245 (ZS), and 152,766,648 (H7) pairs were valid Hi-C data, thus accounting for 51.62, 73.49, and 95.48% of the uniquely correlated data, respectively, as detected by Hi-C-Pro in the Hi-C dataset (Supplementary Tables S1–S3). Overall, our evaluation indicates that the quality of Hi-C data of all three varieties is high. Among the three varieties, the quality of Hi-C data showed the following order: H7 > ZS > YH. Only valid read pairs were used for subsequent analyses.

Prior to constructing the chromosomal-level genome assembly, the initial Hi-C data-based assembly was corrected using BWA-MEM. Contigs were broken into 50-kb fragments, and sequences that could not be located on the original assembly were reassembled using Hi-C as candidate error regions. Then, to complete error correction of the initial assemblies, the locations of low Hi-C coverage depths in these regions were identified as error points. After correction, the genome was assembled using LACHESIS. After the Hi-C assembly and manual adjustment, genome sequence lengths of the three chestnut varieties, 671.99 Mb (YH), 790.99 Mb (ZS), and 678.90 Mb (H7), were located on 12 chromosomes, accounting for 98.84, 100, and 98.79% of the genome sequence length, respectively (Supplementary Tables S4–S6).

A total of 995 (64.57%) sequences mapped to YH, 1014 (100%) to ZS, and 927 (62.76%) to H7. Finally, the genomes of YH, ZS, and H7 assembled by Hi-C were analyzed, and the contig N50 and scaffold N50 values were determined as follows: 3.61 and 50.50 Mb, respectively, for YH; 1.69 and 65.05 Mb, respectively, for ZS; and 3.18 and 52.16 Mb, respectively, for H7 (Supplementary Tables S7–S9).

**Figure 1:**
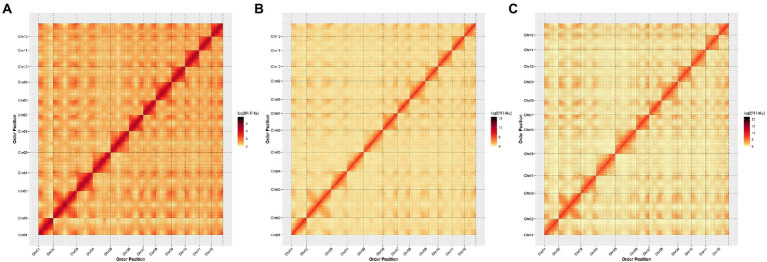
Hi-C interaction heat maps showing interactions among 12 chromosomes of each Chinese chestnut variety with the bin size of 10 kb resolution, using ggplot2 in the R package to evaluate the quality of the chromosomal-level genome assembly. **(A)**: YH; **(B)**: H7; and **(C)**: ZS.

To better compare the quality of the three chromosome-level genome assemblies, we generated a genome-wide Hi-C heat map for each variety. All heat maps showed a distinction among the 12 chromosome groups. Within each group, the intensity of the interaction was the strongest along the diagonal (i.e., between adjacent sequences on the chromosome), while that between distant sequences was weak. This agrees with the principles of Hi-C auxiliary genome assembly, and shows that our genome assembly is high quality (Figure 1).

### Completeness of the assembled genome

The three short sequences of Chinese chestnut genome obtained using the Illumina HiSeq platform were compared with the reference genome using the BWA software, and over 98.15% of the clean reads could be mapped to contigs. The CEGMA database, which contains 458 conserved core eukaryotic genes (CEGs), was used to assess the integrity of the final genome assembly (Table 2).

**Table 2:** Assessment of the integrity of core genes in the three Chinese chestnut varieties.

Variety	No. of 458 CEGs present in the assembly	Percentage of 458 CEGs present in the assembly	No. of 248 highly conserved CEGs present	Percentage of 248 highly conserved CEGs present
YH	422	92.14%	204	82.26%
ZS	438	95.63%	214	86.29%
H7	426	93.01%	202	81.45%

Finally, 90.00% (YH), 95.00% (ZS), and 90.97% (H7) of complete BUSCOs were found in the assemblies (Table 3). This indicates that all three genome assemblies are relatively complete and of high quality.

**Table 3:** Assessment of BUSCO notations in the *C. mollissima* genomes.

	YH	ZS	H7
Complete BUSCOs (C)	1,296 (90.00%)	1,368 (95.00%)	1,310 (90.97%)
Complete and single-copy BUSCOs (S)	1,244 (86.39%)	1,257 (87.29%)	1,262 (87.64%)
Complete and duplicated BUSCOs (D)	52 (3.61%)	111 (7.71%)	48 (3.33%)
Fragmented BUSCOs (F)	27 (1.88%)	25 (1.74%)	28 (1.94%)
Missing BUSCOs (M)	117 (8.12%)	47 (3.26%)	102 (7.08%)
Total Lineage BUSCOs	1,440	1,440	1,440

### Evaluation of genome collinearity

**Figure 2:**
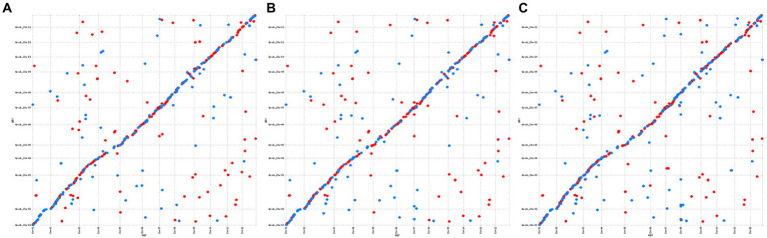
Analysis of collinearity between *C. mollissima and Q. robur* genomes using the MUMmer software. **(A)**: YH; **(B)**: H7; and **(C)**: ZS.

*C. mollissima* and *Q. robur* are two related Fagaceae species that carry an identical number of chromosomes and exhibit high genome sequence similarity. Therefore, we compared the genomes of these two species to verify the accuracy of the three *C. mollissima* genome sequences. The results revealed high degree of synteny between homologous chromosomes of the two species, and further confirmed the reliability of our new genome assemblies (Figure 2).

### Repeat annotation, gene prediction, and gene annotation

In YH, ZS, and H7 genomes, 437.75, 423.16, and 442.76 Mb repeat sequences were discovered, accounting for 64.38, 53.49, and 64.43% of the assembled *C. mollissima* genomes, respectively. The predominant repeat types were Gypsy, Copia, Lard, Line, and unknown (Supplementary Tables S10–S12).

Using a combination of *ab initio*-, homology-, and RNA-seq-based methods, a total of 31,792, 32,012, and 32,411 protein-coding genes were predicted in YH, ZS, and H7 genomes, respectively, with an average gene length of 4,523.08, 5,229.36, and 4,525.15 bp, respectively (Supplementary Table S13).

The non-coding RNA prediction identified 136 miRNAs, 483 rRNAs, and 641 tRNAs in YH; 152 miRNAs, 383 rRNAs, and 659 tRNAs in ZS; and 152 miRNAs, 571 rRNAs, and 740 tRNAs in H7 (Supplementary Table S14).

Next, we examined pseudogenes, which are similar to functional genes in terms of their nucleotide sequence but have evolved a novel function because of a mutation, such as insertion or deletion. Based on GeneWise, a total of 1921, 2,199, and 2009 pseudogenes were identified in YH, ZS, and H7 genomes, respectively, with an average length of 2903.33, 3940.76, and 2682.38 bp, respectively. Finally, 91.50% (YH), 97.43% (ZS), and 91.74% (H7) of the genes were successfully annotated based on existing databases; the functional classifications of these genes are summarized in Supplementary Table S15.

### Comparative genome analysis

Genome sequences of the three Chinese chestnut varieties were compared with those of nine related plant species using OrthoMCL. A total of 20,622, 21,053, and 19,756 gene families and 77, 41, and 102 unique gene families were discovered in YH, ZS, and H7, respectively (Supplementary Table S16).

**Figure 3:**
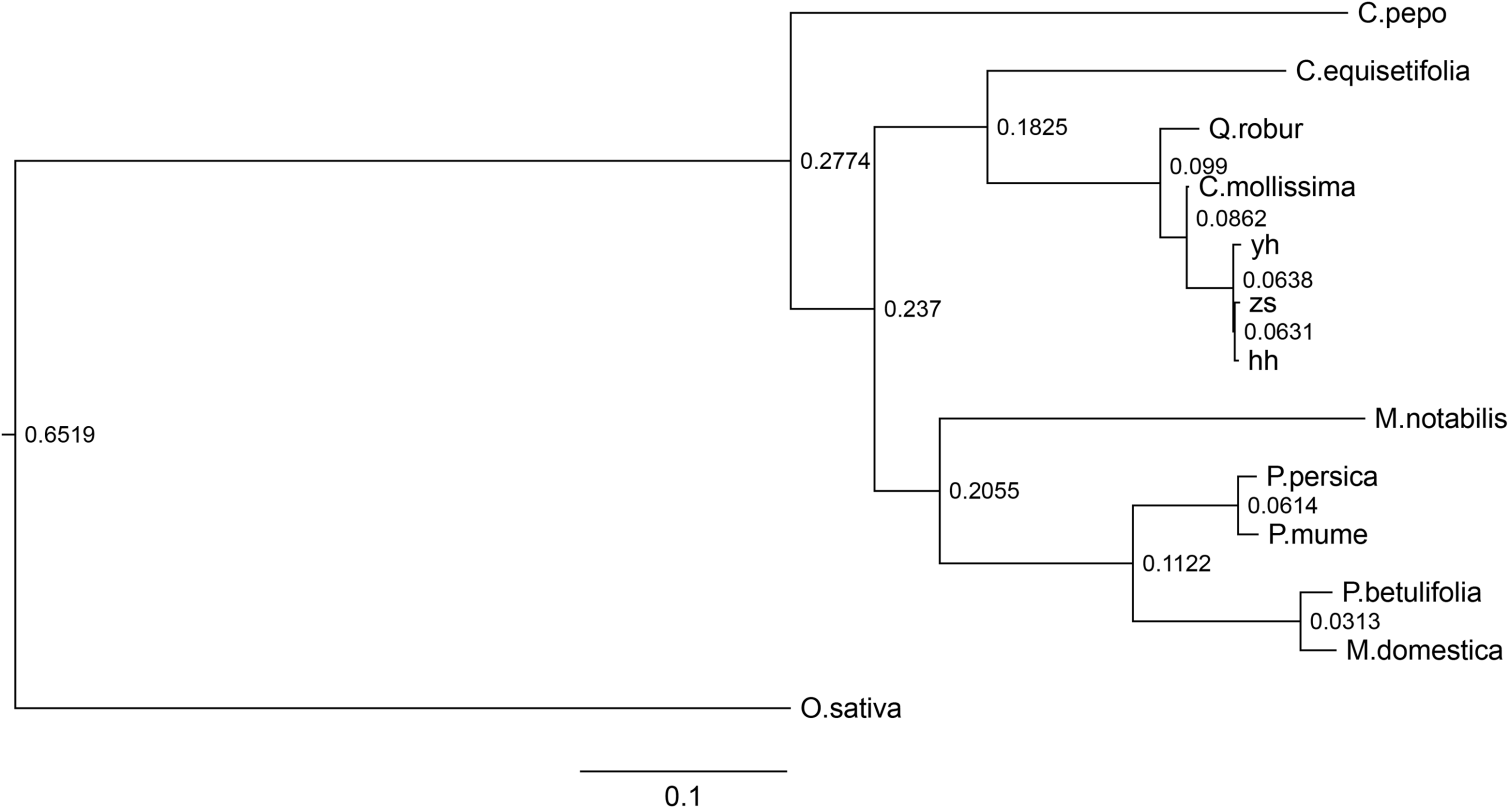
Analysis of evolutionary relationships among Chinese chestnut and nine other species. The phylogenetic tree was constructed by PHYML software using the single-copy protein sequences.

Compared with other plant species, Chinese chestnut varieties contain fewer unigene families. To further understand the evolutionary relationship between Chinese chestnut and other related plant species, PHYML was used with a combination dataset of the protein sequences of single-copy genes of Chinese chestnut and nine other species, and a phylogenetic tree was constructed using the maximum likelihood method. The results supported the hypothesis that Chinese chestnut and oak are sister groups (Figure 3).

### Pan-genome analysis of three Chinese chestnut varieties

Alignment analysis by MUMmer software revealed that all 12 chromosomes in ZS were larger than those in the other two cultivars, especially chromosomes 2, 4, 5, and 8, and some fragments from YH and H7 together formed the chromosomal segments of ZS (Supplementary Figure S2).

**Figure 4:**
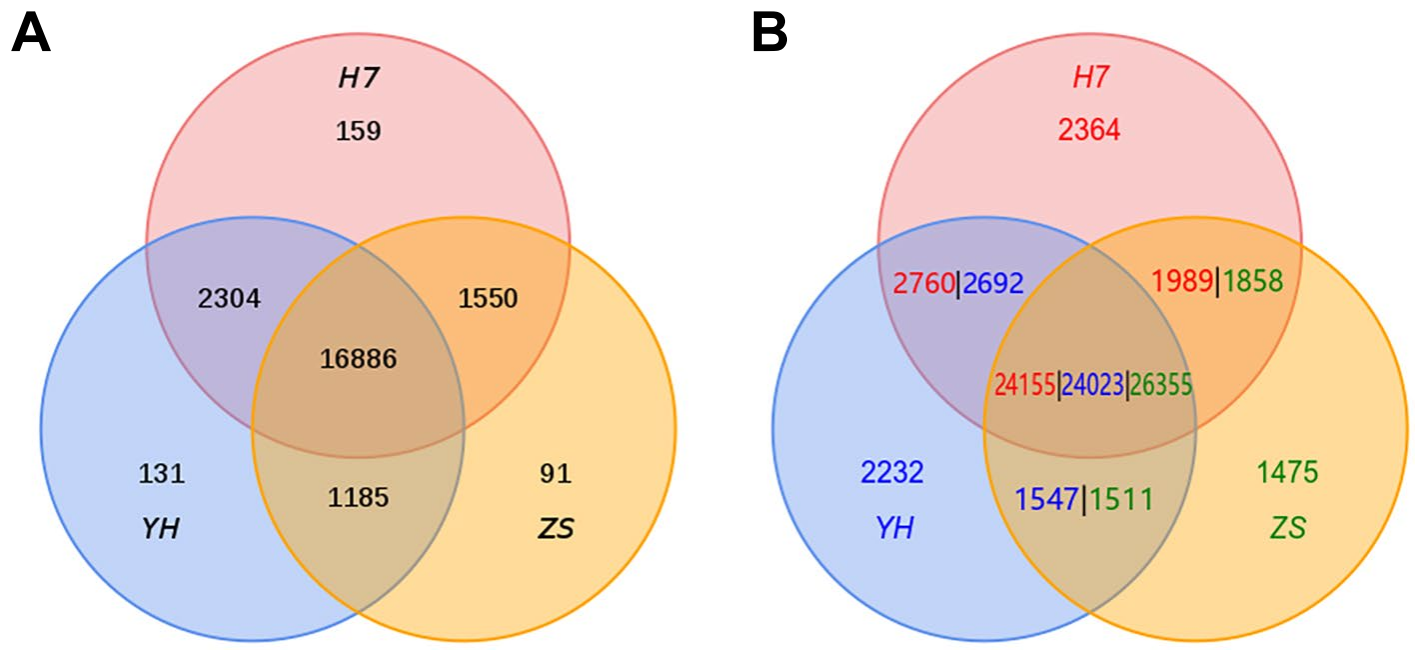
Venn diagram of the number of homologous gene families in H7, YH, and ZS genomes **(A)** and the number of homologous genes in H7, YH, and ZS genomes **(B)**, results from PAV analysis.

Through the identification of homologous genes and cluster analysis of gene families, we found 159, 131, and 91 unique gene families in H7, YH, and ZS genomes, respectively, and a total of 13,248 single-copy direct homologous genes in the three chestnut varieties. Based on the results of gene family identification, the number of PAVs in the three genomes was calculated, and a total of 2,364, 2,232, and 1,475 unique genes were identified in H7, YH, and ZS genomes, respectively (Figure 4; Supplementary Table S18).

### *Castanea mollissima* waxy gene (*GBSS II*) family analysis

Four *GBSS II* gene family members were identified in *C. mollissima* genomes, based on the original annotation, but were later confirmed as three genes based on manual correction after motif and domain analyses. The nucleotide sequences of waxy genes and the corresponding amino acid sequences are shown in Supplementary material chestnut-waxy-gene.pdf. To view the corrected gene structure annotation, see Supplementary material FixWaxy.gff3.

**Figure 5:**
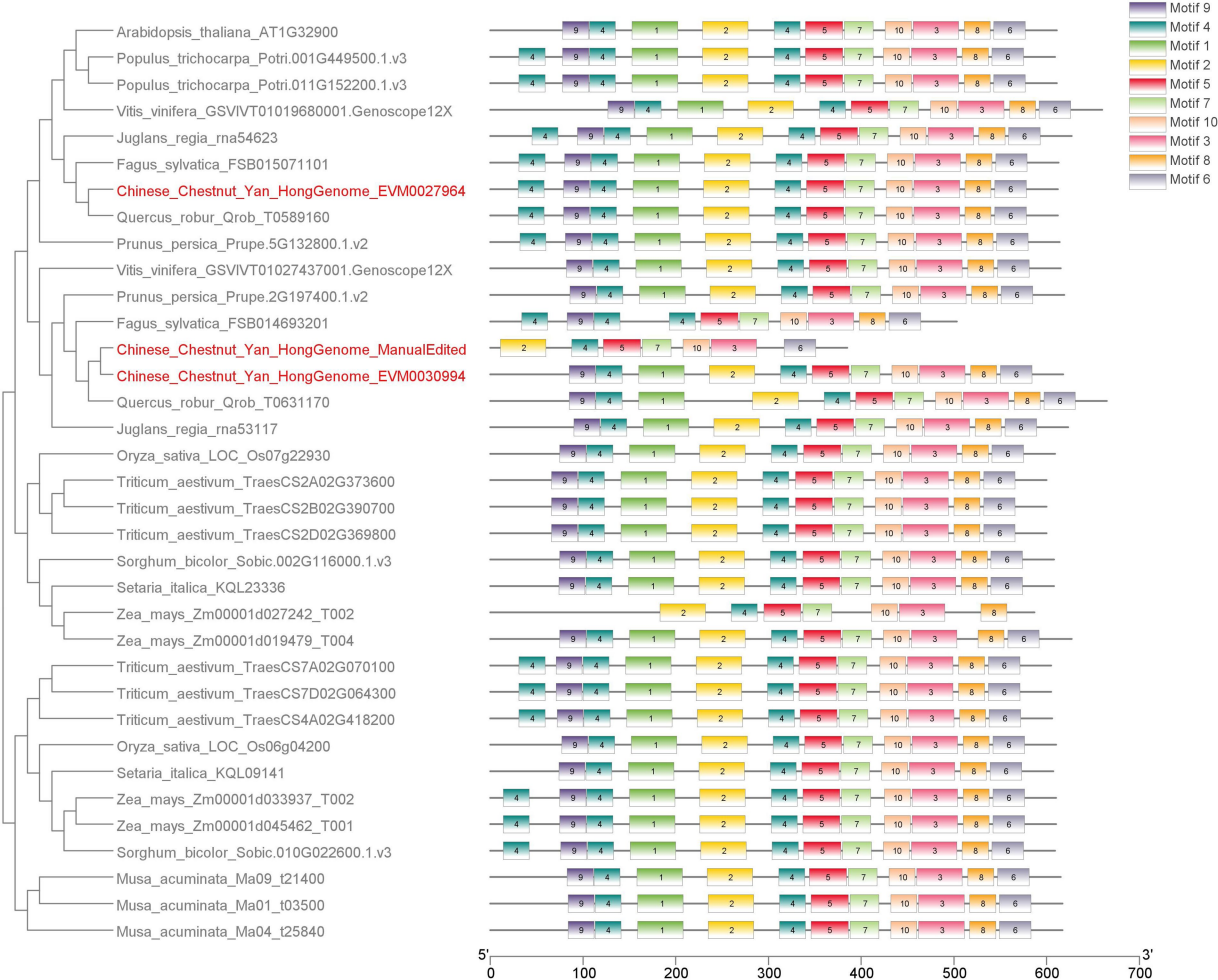
Evolution tree and motifs analysis of Chestnut GBSS II family members and 32 orther species. Phylogenetic trees were constructed using IQtree software.

Did the waxy (*GBSS II*) gene family undergo expansion in chestnut? To answer this question, we conducted phylogenetic analyses of all GBSS proteins and related family members (Supplementary material chestnut-waxy-gene.pdf). The phylogenetic tree showed a clear GBSS clade. Based on the results of evolutionary analysis, we concluded the following: (1) GBSS I family exists only in monocotyledons; (2) GBSS II family exists in both monocotyledons and dicotyledons; (3) GBSS II in dicotyledons can be divided into two branches, and most species have only one GBSS II-b member in each branch; and (4) GBSS II-b branch in chestnut contains one more member than that in the nearest near source species (Figure 5). Gene structure annotation information in IGV revealed the proximity of the two *GBSS II* genes on chromosome 8 within a 14-kb region (Supplementary material chestnut-waxy-gene.pdf), indicating that the *GBSS II* gene family underwent tandem duplication in chestnut.

### Database construction

**Figure 6:**
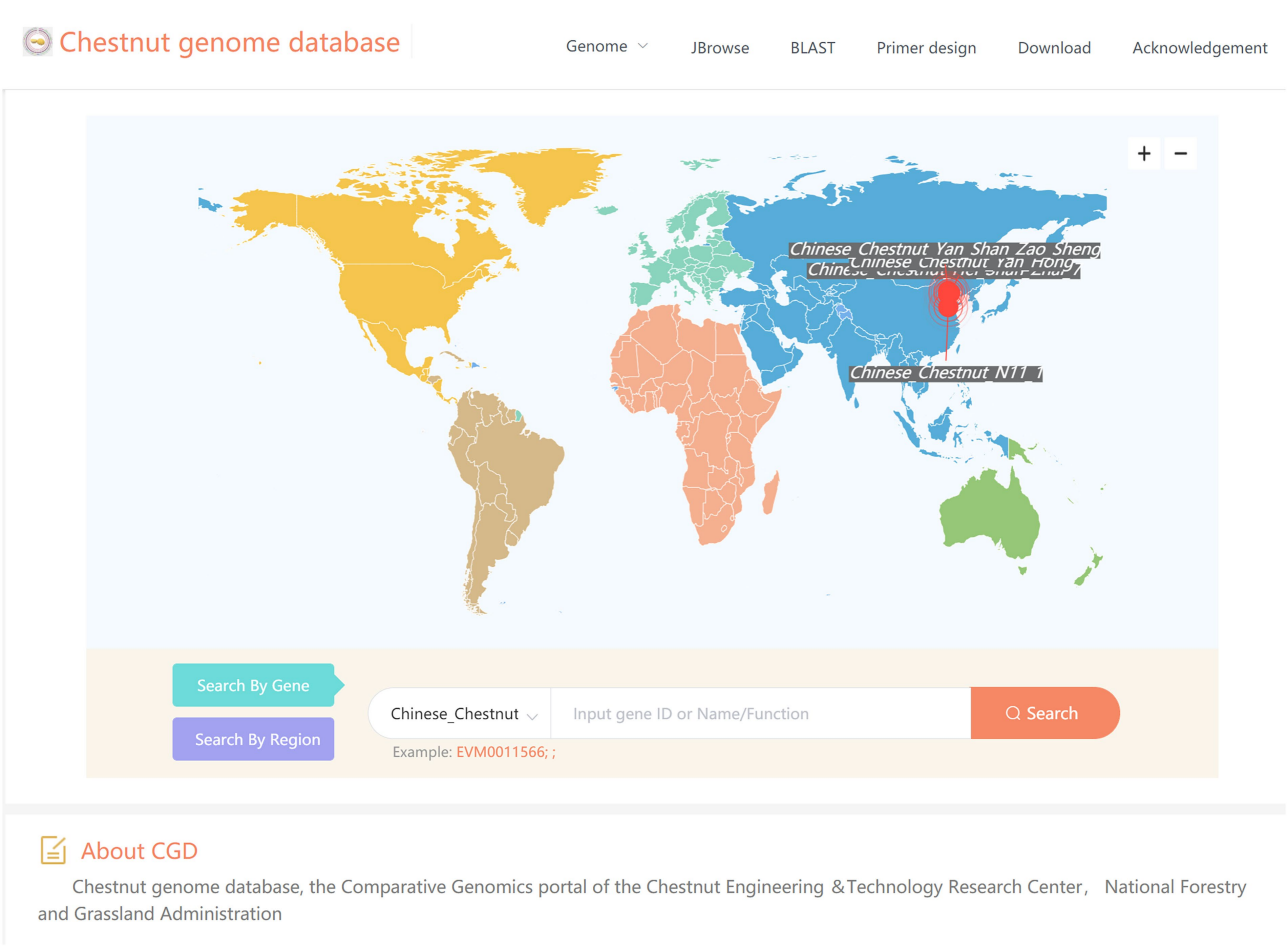
The user interface of Chestnut Genome Database for browsing genomes, searching for homologous sequences and designing primers.

The recent increase in genome resources has produced a wealth of data for in-depth analyses of the biology and evolution of *Castanea* plants, but obtaining and using these resources remains difficult. Therefore, we constructed the Chestnut Genome Database (See Footnote 1). The genomic data of four chestnut varieties, H7, YH, ZS, and N11-1 (Wang et al., 2020a), have been included in this database. This database provides tools for browsing genomes (JBrowse), searching sequence databases (BLAST), and designing primers, combined with GO annotation and KEGG annotation. To better serve the research community, we continue to update our database and develop new tools (Figure 6).

## Discussion

The number of people facing hunger is expected to increase significantly due to continued climate change and the COVID-19 pandemic. In 2020, at least 720million people (≥ 9.9% of the global population) will face hunger; It is the largest percentage of the total population since 2010 (FAO, IFAD, UNICEF, WFP, and WHO, 2021). In order to alleviate global hunger, more attention needs to be paid to non-staple food crops (Chapman et al., 2022). Chestnut, as a tree species that has been used to fight against hunger in history (Gabriele et al., 2020), should be paid more attention and studied.

### Genome size variation

Genome size variation is a fundamental biological characteristic; however, its evolutionary causes and consequences are the topic of ongoing debate (Blommaert, 2020). There are few examples of intraspecific genome size variation and its phenotypic effects. Causes and consequences of genome size variation are particularly well understood in maize, with a recent study finding that genome size was selected for *via* its effects on flowering time at different altitudinal clines, which is consistent with the nucleotypic hypothesis (Bilinski et al., 2018).

In this study, based on the distribution of 21-mers among the Illumina HiSeq reads. The genomes of *C. mollissima* were estimated to be 664.89 Mb (YH), 628.90 Mb (H7) and 752.70 Mb (ZS), with approximately 0.98% (YH), 1.05% (H7) and 0.60% (ZS) heterozygosity. The k-mer distribution curve peaked at a depth of 57 (zs), 51 (YH) and 58 (H7), with a k-mer number of 34,316,017,419 (YH), 36,619,119,572 (H7) and 43,087,876,811 (ZS; Supplementary Figure S1). The total lengths of the genome sequences were determined to be 679.87 Mb with a contig N50 of 3.65 Mb (YH), 790.99 Mb with a contig N50 of 2.17 Mb (ZS), and 687.24 Mb with a contig N50 of 3.39 Mb (H7). After the Hi-C assembly and manual adjustment, genome sequence lengths of the three chestnut varieties, 671.99 Mb (YH), 790.99 Mb (ZS), and 678.90 Mb (H7), were located on 12 chromosomes, accounting for 98.84, 100, and 98.79% of the genome sequence length, respectively. Alignment analysis by MUMmer software revealed that all 12 chromosomes in ZS were larger than those in the other two cultivars, especially chromosomes 2, 4, 5, and 8, and some fragments from YH and H7 together formed the chromosomal segments of ZS (Supplementary Figure S2). The genome of early maturing variety ZS is significantly larger by approximately 100 MB than that of the other two varieties (YH and H7). The fruits of ZS matured one month earlier than the other two. Although genome size was found to be related with flowering time in maize (Bilinski et al., 2018), there is no direct evidence that genome size is related with fruit maturity in chestnut. Through more traditional evolutionary experiments and new techniques, it becomes more clear to understand the basis of intraspecific genome size variation and its potential direct phenotypic effects, as well as the possible causes of intraspecific genome size variation (Blommaert, 2020).

### Database construction and waxy gene (*GBSS II*) family analysis

After the completion of the genome sequencing, an urgent issue is to share the genome data with the research community immediately, which expands the impact of these valuable sequence data and promotes collaboration. However, among the hundreds of sequenced angiosperm genomes, only a few of them have a well-constructed customized database to host its various genome information. A good genome database should meet two criteria: (i) integration of various types of genomic data, and (ii) providing genome analysis tools (Chen et al., 2018). The recent increase in genome resources has produced a wealth of data for in-depth analyses of the biology and evolution of *Castanea* plants, but obtaining and using these resources remains difficult. Therefore, we constructed the Chestnut Genome Database (See Footnote 1). The genomic data of four chestnut varieties, H7, YH, ZS, and N11-1 (Wang et al., 2020a), have been included in this database. This database provides tools for browsing genomes (JBrowse), searching sequence databases (BLAST), and designing primers, combined with GO annotation and KEGG annotation. For an example, we took full advantage of the convenience provided by this database in the waxy gene (*GBSS II*) family analysis.

Starch is one of the most important components of a chestnut, and accounts for 50–80% of its dry matter content (Liu et al., 2015). Chestnut starch is considered as a potentially functional component of dietary fiber, which may be sources of resistant starch, thus improving health (Liu et al., 2022). Chestnut starch has unique physicochemical properties, such as high swelling power, freeze–thaw stability, pasting viscosity, and low gelatinization temperature (Liu et al., 2015, 2019). The characteristics of chestnut starch vary greatly with the variety and its geographical distribution (Long et al., 2018). Waxiness is one of the most important edible qualities of chestnuts; however, this trait also varies greatly with the genotype and production area. Chestnut kernel starch consists mainly of two fractions, amylose and amylopectin. The proportion of amylopectin and amylose in chestnut kernel starch varies among cultivars; the percentage of amylopectin relative to the total starch in a chestnut ranges from 67 to 82%, and the proportion of amylopectin in chestnut kernel starch is approximately 2–5 times that of amylose (Liang, 2011). However, there are few reports on waxy genes in chestnut. Did the waxy (*GBSS II*) gene family undergo expansion in chestnut? To answer this question, we conducted phylogenetic analyses of all GBSS proteins and related family members. Our results suggested expansion of the *GBSS II*-b gene family member in chestnut (relative to the nearest source species). To elucidate the waxiness of Chinese chestnut, it is necessary to combine genome, transcriptome and metabolome studies (Zhang et al., 2015; Chen et al., 2017; Liu et al., 2020). The study of waxy genes in chestnut has enlightenment for the study of other starchy plants.

### Pan-genome analysis and strategy for pyramiding breeding

The high degree of genomic variation observed among individuals belonging to the same species led to the realization that single reference genome do not represent the diversity within a species (Bayer et al., 2020). China is considered a gene center for the genus *Castanea* (Vavilov, 1952; Zhang et al., 2015). Over 300 cultivars have been selected for nut production, which are widely distributed in areas 370–2,800 m above the sea level in China (Li et al., 2009). Obviously, single reference genome cannot meet the needs of Chinese chestnut industry research and development. The resources of crop pan-genomes rather than single reference genomes will accelerate molecular breeding (Golicz et al., 2016a,b; Bayer et al., 2020; Jensen et al., 2020; Murukarthick et al., 2021). However, for some species, pan-genome-assisted breeding efforts remain limited due to the small size of the research communities (Rafael et al., 2021). At present, there are few reports about pan-genome in the study of nut crop. We have overcome various difficulties and carried out pan-genome analysis in chestnut research for the first time.

A pan-genome project should select genotypes that have played an important role in breeding and genetics (Yu et al., 2008; Jain et al., 2019; Schreiber et al., 2020) to maximize the benefits for the research and breeding community. In the present study, we selected three main varieties, namely Hei-Shan-Zhai-7 (drought-resistant variety), Yan-Hong (easy-pruning variety), and Yan-Shan-Zao-Sheng (early-maturing variety), using Oxford Nanopore Technology (ONT) and Illumina HiSeq X sequencing and Hi-C mapping, performing a pan-genome analysis.

Early-maturing chestnut varieties could be put on the market earlier, which would greatly improve the overall value of the nut; chestnut orchards are mostly in mountainous areas with poor irrigation conditions; labor shortage and aging phenomenon in chestnut planting industry are serious (Ren and Jia, 2014; Zhao and Zhang, 2015), therefore, pyramiding breeding of early-maturing cultivars that are drought-resistant and easy-pruning is a priority for chestnut industry. Although several early maturing varieties (e.g., ZS) have been developed, however, genes responsible for early maturity in chestnut have not been investigated to date. Chinese chestnut is a monoecious plant, and having too many male flowers on an individual plant results in the overconsumption of nutrients and water (Feng, 1995). The mutant varieties (e.g., H7) with extremely short catkins has a significantly reduced number of flowers in the male inflorescence, which saves nutrition and water and improves drought resistance (Feng et al., 2011). Other studies have found genes that play important roles in flower development (Dong et al., 2017; Tian et al., 2018; Chen et al., 2019). We have acquired an invention patent- “open pollination” molecular chestnut breeding system (Hu et al., 2017) based on the character of extremely short catkins in H7. However, there is no short-catkin variety bred by molecular marker assisted selection. Only a few varieties (e.g., YH) can keep the number of fruiting branches after extensive cutting back pruning (Fan et al., 2009), the molecular mechanism still unknown.

In this study, based on the results of gene family identification, the number of PAVs in the three genomes was calculated, and a total of 2,364, 2,232, and 1,475 unique genes were identified in H7, YH, and ZS genomes, respectively (Figure 4; Supplementary Table S18). Based on the pan-genome analysis results, we have formulated the following strategy for pyramiding breeding. According to the pan-genomic research results, combined with the “open pollination” molecular breeding system of Chinese chestnut, which saves time and effort, H7, YH and ZS are crossed with each other, and the hybrid fruit is directly optimized. The hybrid fruits containing at least two cultivars’ unique genes will be selected, and the hybrid fruits without unique gene will be discarded. This strategy should accelerate the pyramiding breeding process of early-maturing, drought-resistant and easy-pruning cultivars.

## Conclusion

In this study, we constructed high-quality chromosome-level genome assemblies of three *C. mollissima* varieties using a combination of ONT sequencing, Illumina HiSeq X sequencing, and Hi-C mapping. We constructed the chestnut genome database which provides tools for browsing genomes (JBrowse), searching sequence databases (BLAST), and designing primers. Through the identification of homologous genes and the cluster analysis of gene families, we found that H7, YH and ZS had 159,131and 91 unique gene families, respectively. The Presence/Absence variations (PAVs) information of the three sample genes was calculated, and there were 2,364, 2,232, and 1,475 unique genes in H7, YH and ZS, respectively. Our results suggested expansion of the *GBSS II-b* gene family member in chestnut (relative to the nearest source species). The pan-genome analysis of three main chestnut varieties will provide a solid foundation for future trait improvement, seedling breeding, conservation, and phylogenetic research.

## Data availability statement

The datasets presented in this study can be found in online repositories. The names of the repository/repositories and accession number(s) can be found in the article/Supplementary material.

## Author contributions

GH conducted the experiments, analyzed the data, and prepared the manuscript. LC, YC, WM, YQ, and YL performed the collection and processing of samples and analyzed the data. YL coordinated the experiments. All authors contributed to the article and approved the submitted version.

## Funding

This work was financially supported by the National Basic Research Program of China (Grant no. 2013FY111700-2), the China National Key R&D Program (Grant no. 2018YFD1000605), the Special Fund for the Construction of Scientific and Technological Innovation Capability (Grant nos. KJCX20200114 and PT2022-07), Presidential Foundation of Institute of Forestry and Pomology (Grant no. LGY201901) and the Youth Scientist Fund of Institute of Forestry and Pomology, Beijing Academy of Agriculture and Forestry Sciences (LGYJJ202007).

## Conflict of interest

The authors declare that the research was conducted in the absence of any commercial or financial relationships that could be construed as a potential conflict of interest.

## Publisher’s note

All claims expressed in this article are solely those of the authors and do not necessarily represent those of their affiliated organizations, or those of the publisher, the editors and the reviewers. Any product that may be evaluated in this article, or claim that may be made by its manufacturer, is not guaranteed or endorsed by the publisher.

## Footnotes

^2^ https://github.com/ruanjue/wtdbg

^3^ http://transdecoder.github.io
