# Supplementary material for: Pan-genome analysis of three main Chinese chestnut varieties
Source: Front Plant Sci. 2022 Jul 25;13:916550. doi: 10.3389/fpls.2022.916550 (PMC9358723; doi:10.3389/fpls.2022.916550)
Supplement: Supplementary file 2 [file Data_Sheet_2.PDF]

Chestnut-waxy-gene Supplementary materials

1.Sequences:

>Chinese_Chestnut_Yan_HongGenome_EVM0027964
MATVTTSHFVSSSHVNCHGTSGSESKVNFGLRSQAMTHNGLRLLNKLDILKMRTQTKAIA
RPARSTVSKTEKDRPNGKIVCEQGMNLVFVGAEIGPWSKTGGLGDVLGGLPPAMAAKGHR
VMTVSPRYDQYKDAWDTSVLVEMKVGDRIETVRFFHCYKRGVDRVFVDHPTFLEKVWGKT
GSKIYGPRAGLDYEDNQLRFSLLCQAALEAPRVLNLKSNEYFSGPYGEDVIFIANDWHTA
LLPCYLKTMYKSRGLYKNAKVAFCIHNIAYQGRFPFSDFSLLNLPDQFKSSFDFIDGYEK
PVKGRKINWMKGGIIESDRVLTVSPYYAQELVSGIDKGVELDNIIRKTGITGIVNGMDVQ
EWNPSRDKYIDIKYDSTTVMDAKPLLKEALQAEVGLPVDQNIPLIGFIGRLEEQKGSDIL
VEAISKFIGQDVQIIILGTGKKPMEKQIEQLEVLYPNSARGVAKFNVPLAHMVIAGADFM
LIPSRFEPCGLIQLHAMRYGTVPIVASTGGLVDTVKEGFTGFHMGAFNVECDAVDPADVT
AVATNVKRALATFGTPVLKEMIQNCMAQDFSWKGPARQWEKMLLSLDVAGSEPGTEREEI
APLAKENVATP*
>Chinese_Chestnut_Yan_HongGenome_ManualEdited
MYSNRSSKKEWDFYGRLAALEAPRVLALNSNEYFSGPYGEEVAFYIHNIAYQGRFAFTDF
SLLNLPDELKSSFDFIDGYEKPVKGRKINWMKAGILESDKVLTVSPYYAQELFSGEDKGV
ELDNIIRKTGIQGIVNGMDVQEWNPLTDKFTNVKYDASTVLDAKPLLKEALQAEVGLPVD
RNIPVMGFIGRLEEQKGSDILVEAIPHFIKENVQIIVLGTGKKPMEKQLEQLEKIYPDKA
RGVAKFNVPLAHMIIAGADFILVPSRFEPCGLIQLHAMRYGTVPIVASTTGLIDTVKEGF
TGFQMGSFNVEVITHFLSHARAIATYGTPAFTEIIQNCMAQDLSWKGPAKKWEEVLLSLG
VAGSEPGIEGEEIAPLAKENVATP*
>Chinese_Chestnut_Yan_HongGenome_EVM0030994
MATVAGSNFVSTSSRVNYGSVTSGSEAKVALMKFGLINHTVTHSGLRSLNKVDELMHVRT
MAKLTARQTRSKTFKSGNAWTSRTIVCGSGMNLIFVGTEVGPWSKTGGLGDVLGGLPPAM
AANGHRVMTVSPRYDQYKDAWDTDVVIELKVGDKIEKVRFFHCHKRGVDRVFVDHPAFLE
KVWGKTKSKIYGPIAGEDFQDNQLRFSLLCQAALEAPRVLALNSNEYFSGPYGEEVIFIA
NDWHTALIPCYLKTIYKPKGIYSTARVAFCIHNIAYQGRFAFTDFSLLNLPDELKSSFDF
IDGYEKPVKGRKINWMKAGILESDKVLTVSPYYAQELVSGEDKGVELDNILRKTGIQGIV
NGMDVQEWNPLTDKYTNVKYDASTVLDAKPLLKEALQAEVGLPVDRNIPIIGFIGRLEEQ
KGSDILVEAIPHFIKENVQIIVLGTGKKPMEKQLEQLEKIYPDKARGVAKFNVPLAHMII
AGADFILVPSRFEPCGLIQLHAMRYGTVPIVASTGGLVDTVKEGFTGFQMGSFNVECEAV
DPADVTAVATNAKRAIATYGTPAFTEIIQNCMAQDLSWKGPAKKWEEVLLSLGVAGSEPG
IEGEEIAPLAKENVATP*
>Chinese_Chestnut_Yan_HongGenome_EVM0027964
ATGGCAACTGTGACCACCTCACACTTTGTATCCAGTTCACATGTCAATTGCCATGGAACG
TCCGGATCAGAAAGTAAAGTAAACTTTGGCCTAAGGAGTCAAGCAATGACTCATAATGGG
TTAAGGTTGTTGAACAAGCTAGATATACTAAAGATGAGAACCCAAACAAAAGCAATTGCT
AGGCCAGCAAGAAGCACAGTGAGTAAAACTGAGAAGGACAGACCCAATGGGAAAATTGTA
TGTGAACAAGGAATGAACTTGGTGTTTGTGGGAGCTGAGATAGGTCCATGGAGCAAGACT
GGTGGACTTGGTGATGTTCTTGGAGGACTTCCACCTGCAATGGCAGCTAAGGGGCACCGT
GTTATGACTGTGTCTCCACGCTATGACCAATACAAAGATGCATGGGACACAAGTGTTCTA
GTTGAGATGAAAGTTGGGGATAGAATTGAAACCGTTCGCTTCTTCCACTGCTATAAACGA
GGAGTTGATCGTGTATTCGTGGATCACCCAACGTTCCTCGAAAAGGTATGGGGAAAAACT

GGATCCAAAATCTATGGCCCTAGGGCTGGTCTGGATTACGAGGATAACCAGCTGCGATTC
AGCTTGTTGTGCCAGGCTGCTCTGGAGGCACCAAGGGTTCTGAACTTGAAAAGCAATGAA
TATTTCTCCGGACCATATGGGGAGGATGTTATCTTCATTGCCAATGATTGGCACACTGCA
CTTCTTCCATGCTACCTAAAAACAATGTACAAATCAAGGGGACTTTATAAAAATGCCAAG
GTTGCTTTCTGCATTCATAACATAGCTTACCAGGGAAGATTCCCCTTCTCAGACTTTTCA
CTTCTCAATTTGCCTGATCAATTCAAGAGTTCTTTTGACTTTATTGATGGCTATGAGAAG
CCAGTGAAAGGAAGGAAAATCAATTGGATGAAGGGTGGAATAATAGAATCGGACAGGGTC
CTGACTGTGAGCCCATACTATGCCCAGGAACTTGTTTCTGGAATTGACAAGGGTGTGGAA
TTGGATAACATCATTCGTAAGACTGGCATCACTGGTATCGTGAATGGGATGGATGTTCAA
GAGTGGAATCCTTCCAGGGACAAATACATTGATATTAAATATGATTCCACAACCGTTATG
GATGCAAAGCCTCTATTGAAGGAAGCCCTTCAAGCAGAAGTTGGGTTGCCTGTTGACCAA
AACATCCCTTTGATAGGCTTCATTGGAAGACTAGAAGAGCAGAAAGGTTCAGATATTCTA
GTAGAAGCTATTTCAAAGTTCATTGGGCAGGATGTTCAAATTATAATCCTTGGAACTGGC
AAAAAGCCCATGGAGAAGCAAATTGAACAGCTGGAGGTACTATATCCCAACAGTGCCCGG
GGTGTGGCAAAATTTAACGTCCCCTTAGCTCATATGGTGATTGCTGGGGCTGATTTTATG
CTGATCCCAAGTAGATTTGAACCCTGTGGTCTCATTCAGTTACATGCCATGCGATATGGA
ACGGTACCTATTGTTGCCTCAACCGGTGGACTTGTTGATACTGTCAAAGAAGGATTTACA
GGATTCCATATGGGAGCTTTCAATGTTGAATGTGATGCAGTTGATCCAGCTGATGTAACT
GCAGTAGCCACTAATGTCAAGAGAGCTCTTGCAACTTTCGGTACCCCTGTTTTGAAAGAG
ATGATCCAGAATTGCATGGCCCAAGATTTTTCATGGAAGGGACCAGCCAGACAATGGGAA
AAGATGCTGCTGAGCCTGGATGTTGCTGGCAGTGAACCTGGAACTGAAAGGGAGGAGATT
GCTCCTCTTGCCAAGGAAAATGTTGCCACTCCTTGA
>Chinese_Chestnut_Yan_HongGenome_ManualEdited
ATGTATAGTAACCGAAGTAGTAAGAAAGAATGGGACTTCTATGGTAGACTGGCAGCTCTA
GAGGCACCAAGGGTTCTAGCACTTAACAGCAATGAATACTTCTCTGGACCATATGGTGAA
GAGGTTGCCTTTTACATTCACAACATTGCTTACCAAGGCAGATTTGCCTTCACAGATTTC
TCACTTCTCAATCTCCCAGATGAATTGAAAAGCTCTTTTGATTTTATTGATGGGTATGAA
AAGCCAGTTAAGGGAAGAAAAATCAATTGGATGAAAGCTGGAATTTTAGAATCAGACAAG
GTTTTAACCGTGAGCCCATACTATGCCCAAGAACTTTTTTCTGGAGAAGACAAAGGAGTG
GAATTGGATAACATCATTCGTAAAACTGGCATTCAAGGAATTGTGAATGGCATGGATGTC
CAGGAATGGAACCCCTTAACTGACAAATTTACAAACGTCAAATATGATGCTTCAACTGTG
TTGGATGCAAAGCCTCTTTTGAAGGAAGCCCTCCAAGCTGAAGTTGGATTGCCAGTCGAT
AGAAACATCCCTGTCATGGGCTTCATTGGTAGACTTGAAGAGCAGAAAGGTTCAGATATT
CTTGTAGAAGCCATTCCCCATTTTATCAAAGAGAATGTTCAAATAATAGTCCTTGGGACT
GGCAAAAAACCAATGGAGAAGCAGCTTGAACAGCTGGAGAAAATATACCCTGACAAGGCC
CGAGGAGTGGCAAAATTCAACGTTCCCCTGGCTCATATGATAATAGCTGGTGCTGATTTT
ATACTGGTTCCTAGTAGATTTGAGCCCTGTGGTCTCATTCAATTACATGCCATGCGTTAT
GGAACTGTGCCTATTGTTGCCTCAACTACTGGATTAATTGACACGGTTAAAGAAGGCTTC
ACAGGATTTCAAATGGGAAGCTTCAATGTTGAAGTAATAACTCATTTCCTTTCACATGCT
CGGGCCATCGCAACCTATGGAACTCCAGCTTTCACTGAGATAATACAGAATTGCATGGCT
CAAGATCTCTCATGGAAGGGACCAGCTAAGAAGTGGGAGGAGGTGCTGCTAAGTTTGGGG
GTGGCAGGAAGTGAACCTGGAATCGAGGGTGAGGAAATAGCTCCACTCGCAAAGGAAAAC
GTTGCAACACCTTAA
>Chinese_Chestnut_Yan_HongGenome_EVM0030994

ATGGCAACTGTGGCTGGTTCAAACTTTGTATCAACAAGTTCTCGTGTCAACTATGGATCA
GTAACTTCTGGATCAGAGGCTAAAGTAGCACTGATGAAATTTGGATTAATAAATCACACC
GTGACTCACAGTGGGTTAAGATCTTTGAACAAAGTGGATGAACTAATGCATGTCAGAACC
ATGGCAAAATTAACTGCTAGGCAAACCAGGAGTAAAACATTCAAGAGTGGGAATGCATGG
ACTTCACGGACCATTGTTTGCGGAAGTGGGATGAATTTGATCTTTGTGGGGACAGAAGTA
GGTCCCTGGAGCAAAACCGGTGGACTTGGAGATGTTCTTGGAGGTCTGCCACCAGCAATG
GCGGCCAACGGGCATCGTGTTATGACTGTCTCTCCACGTTATGATCAGTACAAAGACGCG
TGGGATACAGACGTTGTAATTGAGCTTAAAGTAGGAGATAAAATCGAAAAGGTTCGCTTC
TTCCATTGCCACAAAAGAGGAGTTGATCGCGTTTTTGTGGATCACCCAGCGTTTCTTGAA
AAGGTGTGGGGGAAAACCAAATCCAAAATTTATGGGCCTATTGCTGGAGAGGATTTCCAG
GACAACCAACTTCGATTCAGCTTATTATGCCAGGCAGCTCTAGAGGCACCAAGGGTTCTA
GCACTTAACAGCAATGAATATTTCTCTGGACCATATGGTGAAGAGGTCATATTCATTGCC
AACGACTGGCACACTGCCTTGATCCCATGCTACCTGAAAACTATATACAAACCCAAAGGC
ATATACAGCACTGCCAGAGTTGCCTTTTGCATTCACAACATTGCTTACCAAGGCAGATTT
GCATTCACAGATTTCTCACTTCTCAATCTCCCAGATGAATTGAAAAGCTCTTTTGATTTT
ATTGATGGGTATGAAAAGCCAGTTAAGGGAAGAAAAATCAATTGGATGAAAGCTGGAATT
TTAGAATCAGACAAGGTTTTAACTGTGAGCCCATACTATGCCCAAGAACTTGTTTCCGGA
GAAGACAAAGGAGTGGAATTGGATAACATCCTTCGTAAAACTGGCATTCAAGGAATTGTG
AATGGCATGGATGTCCAGGAATGGAACCCCTTAACTGACAAATACACAAACGTGAAATAT
GATGCTTCAACTGTGTTGGATGCAAAGCCTCTTTTGAAGGAAGCCCTCCAAGCTGAAGTT
GGATTGCCAGTGGATAGAAACATCCCTATCATAGGCTTCATTGGTAGACTTGAAGAGCAG
AAAGGTTCAGATATTCTTGTAGAAGCCATTCCCCATTTTATCAAAGAGAATGTTCAAATA
ATAGTCCTTGGGACTGGCAAAAAACCAATGGAGAAGCAGCTTGAACAGCTGGAGAAAATA
TACCCTGACAAGGCCCGAGGAGTGGCAAAATTCAATGTTCCCCTGGCTCATATGATAATA
GCTGGTGCTGATTTTATACTGGTTCCTAGTAGATTTGAGCCCTGTGGTCTCATTCAATTA
CATGCCATGCGTTATGGAACTGTGCCTATTGTTGCCTCAACTGGTGGATTGGTTGACACG
GTTAAAGAAGGCTTCACAGGATTTCAAATGGGAAGCTTCAATGTTGAATGTGAAGCTGTT
GATCCTGCCGATGTGACTGCAGTGGCTACAAATGCGAAAAGGGCCATCGCAACCTATGGA
ACTCCAGCTTTCACTGAGATAATACAGAATTGCATGGCTCAAGATCTCTCATGGAAGGGA
CCTGCTAAGAAGTGGGAGGAGGTGCTGCTAAGTTTGGGGGTGGCAGGAAGTGAACCTGGA
ATCGAGGGTGAGGAAATAGCTCCACTCGCAAAGGAAAACGTTGCAACACCTTAA

2.Gene distribution and gene length

GeneID	Chr	Gene Range	mRNA Range	No. Exon	ORF Len
EVM0027964	Chr02	26150031:26155577	26150031:26155577	14	1836
EVM0030994	Chr08	50511551:50515651	50511551:50515651	13	1854
ManualEdited.gene	Chr08	50521016:50523688	50521016:50523688	10	1155

Note： two of the three members are tandem repeats

3.Subcellular localization prediction

	pI	Mw	Localization
EVM0027964	7.94	67564.8	Chloroplast
ManualEdited	5.21	42587.89	Cytoplasmic
EVM0030994	6.66	67692.85	Chloroplast

Note：Two proteins were localized on chloroplasts, and one protein was localized in the cytoplasm.

4. Evolutionary tree

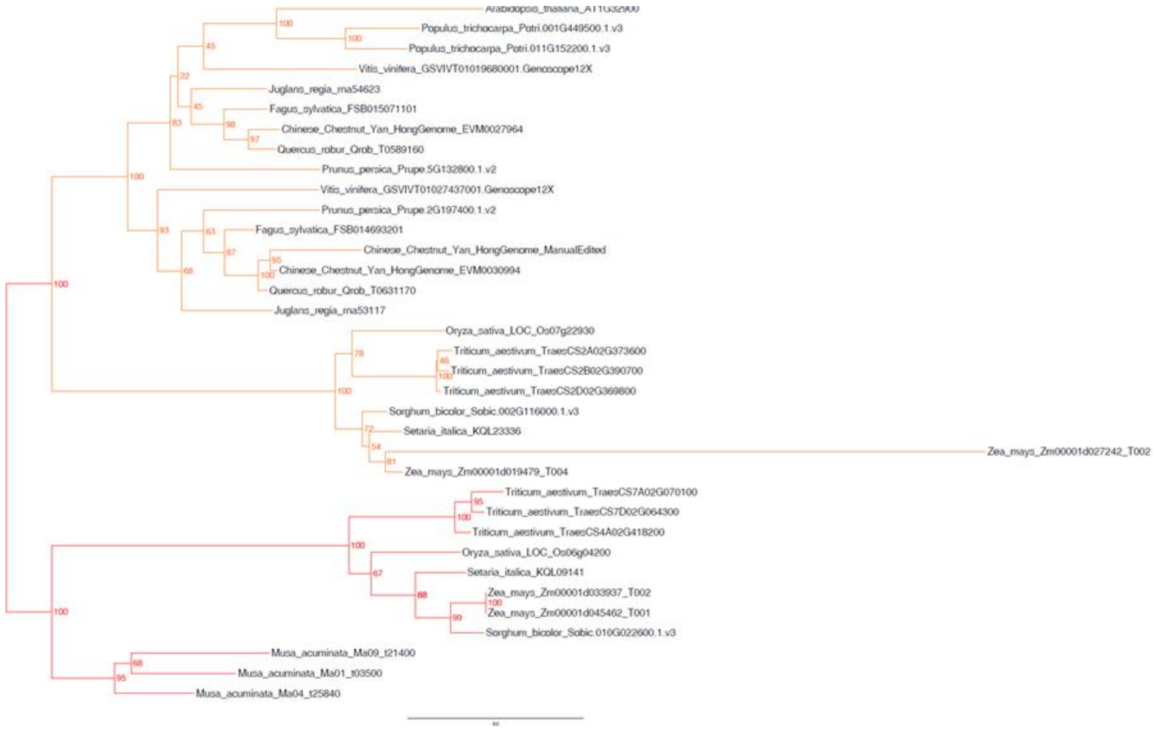

5. Motifs analysis

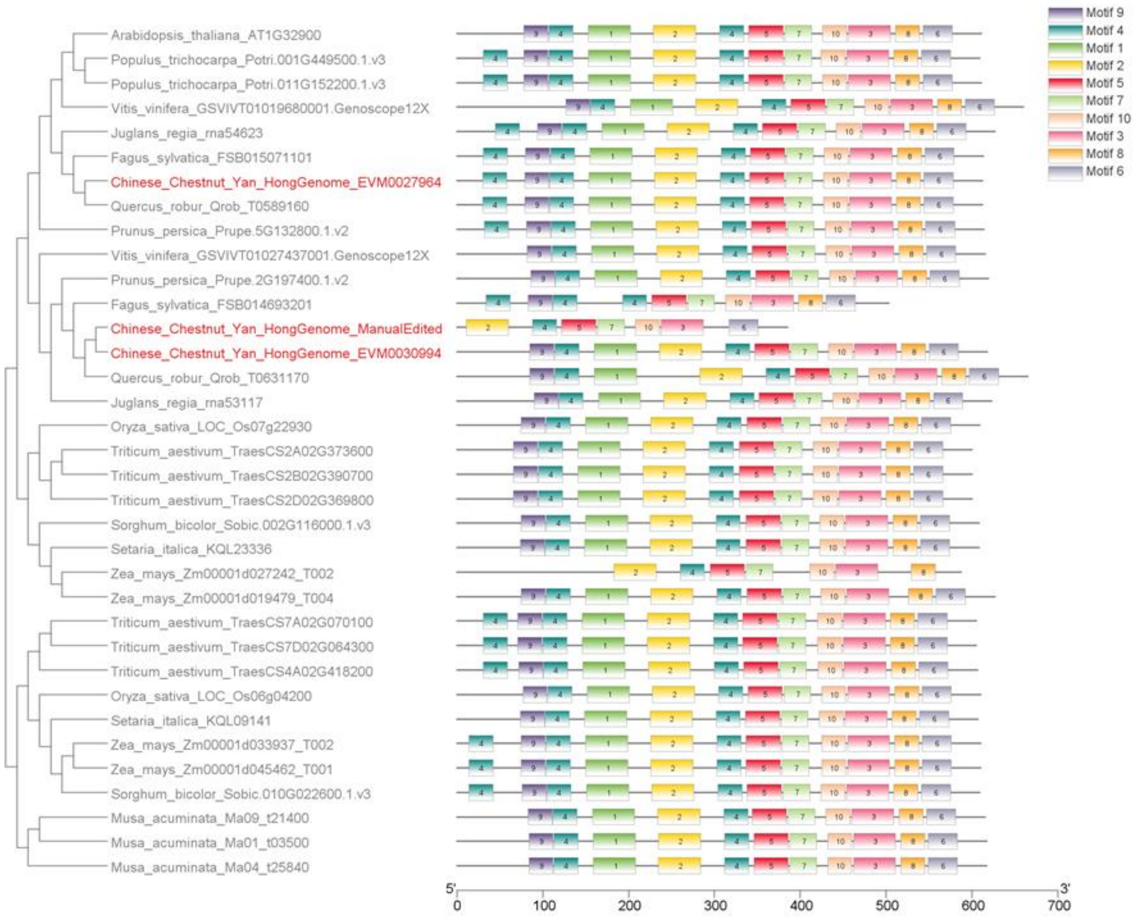

6．Conserved domain prediction

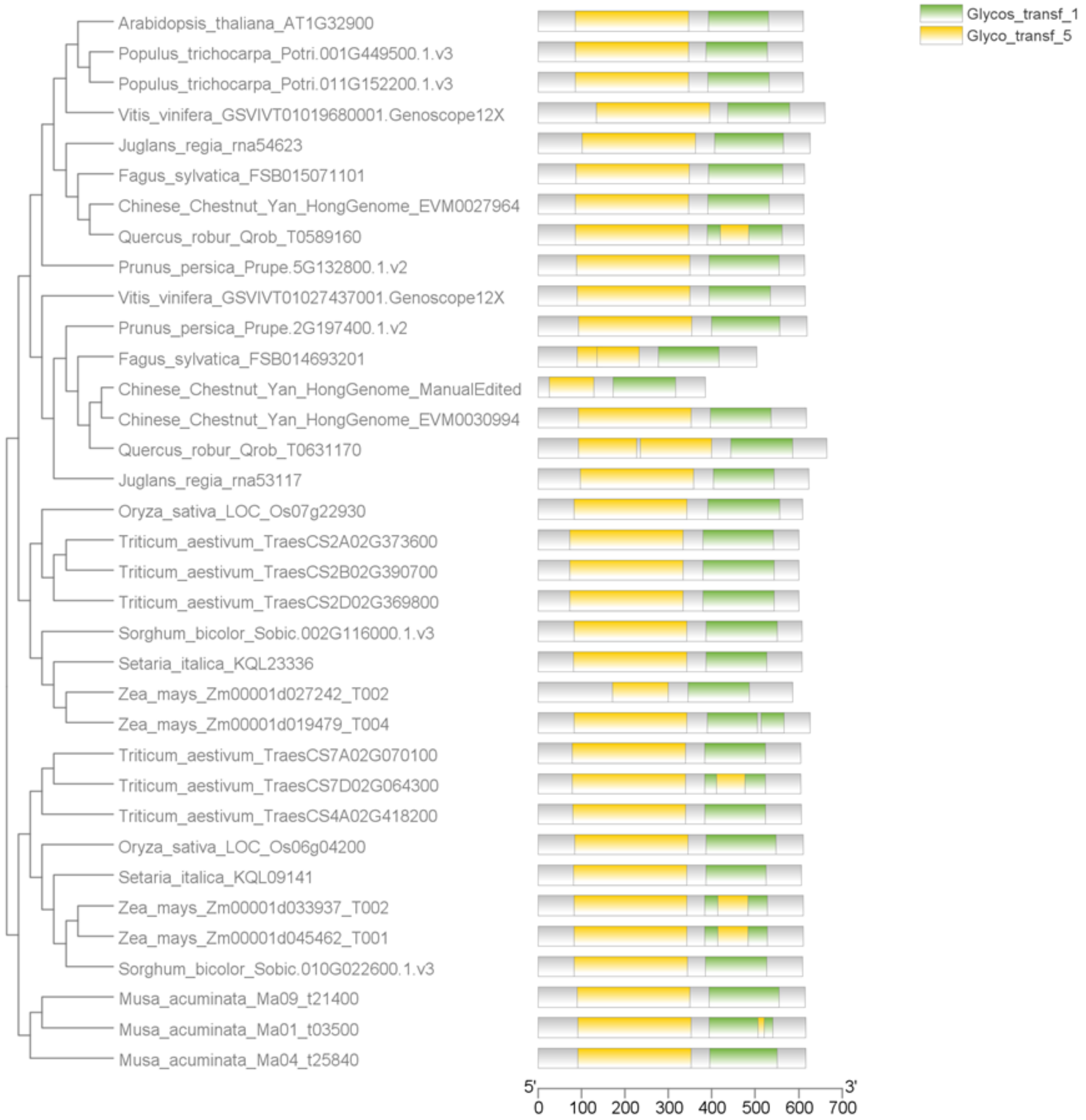

Note： All sequences have the starch synthase catalytic domain Glyco_transf_5 and glycosyltransferase domain Glyco_transf_1 both domains.

7. Is waxy (GBSS II) family expansion present in chestnut?

A clear GBSS clade was identified by phylogenetic tree construction of all GBSS and their close family members, as follows

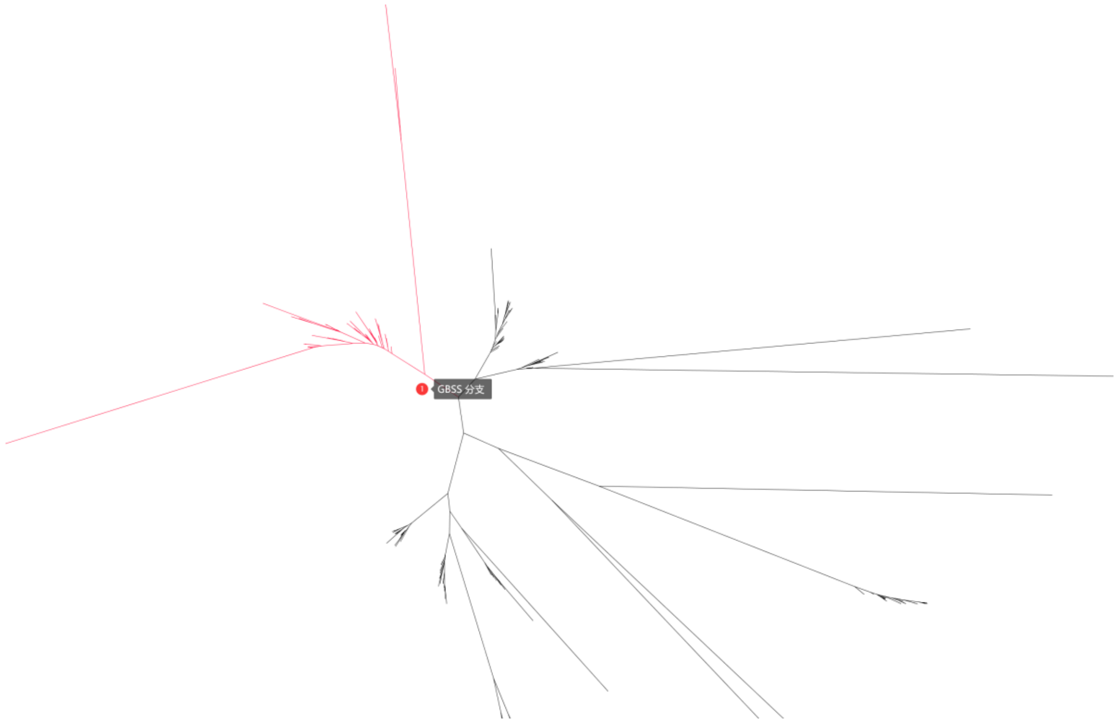

GBSS II in dicotyledons can be divided into two branches, and most species have only one GBSS II-b member in each branch

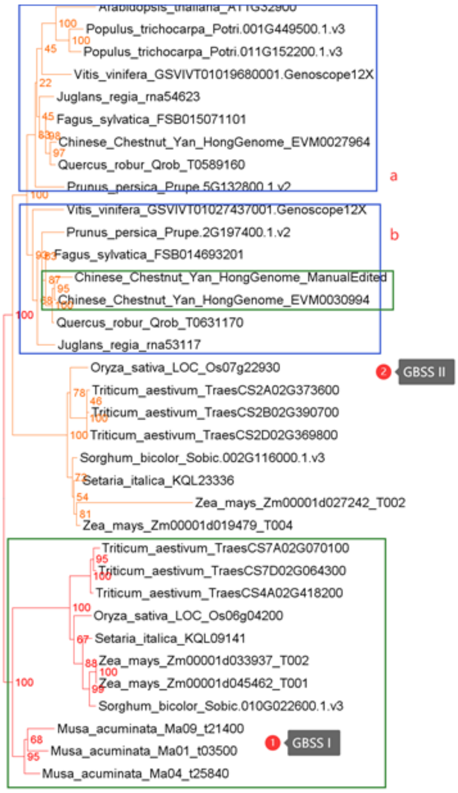

Gene structure annotation information in IGV reveals the proximity of the two GBSS II genes on chromosome 8 within a 14 Kb region, so that the source of the expansion of GBSS II genes in chestnut is tandem duplications.

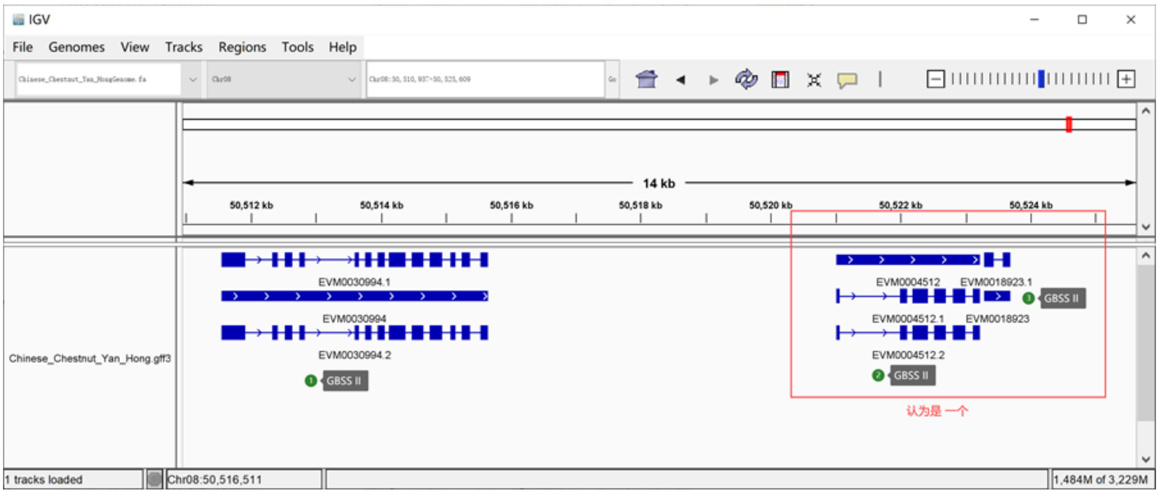
IGV
File Genomes View Tracks Regions Tools Help
14 kb
50,512 kb
50,514 kb
50,516 kb
50,518 kb
50,520 kb
50,522 kb
50,524 kb
Chinese_Chestnut_Yan_Hong.gff3
EVM0030994.1
EVM0030994
EVM0030994.2
GBSS II
EVM0004512
EVM0018923.1
EVM0004512.1
EVM0018923
EVM0004512.2
GBSS II
GBSS II
认为是一个
1 tracks loaded
Chr08:50,516,511
1,484M of 3,229M
