# Supplementary figures and images for: Pan-genome analysis of three main Chinese chestnut varieties
Source: Front Plant Sci. 2022 Jul 25;13:916550. doi: 10.3389/fpls.2022.916550 (PMC9358723; doi:10.3389/fpls.2022.916550)

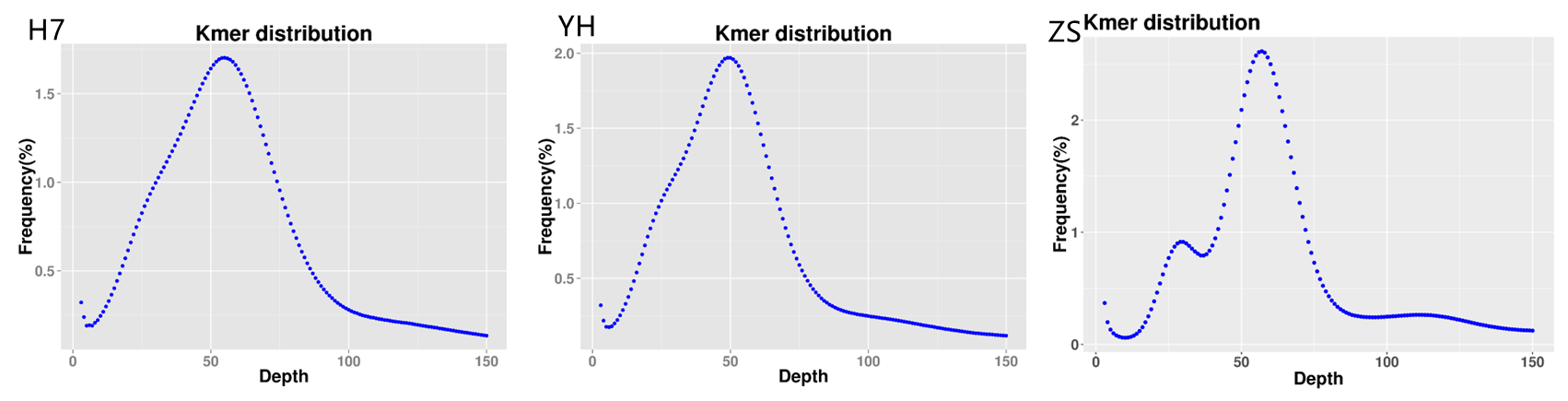

Supplement: Supplementary file 4 [file Image_1.PNG]

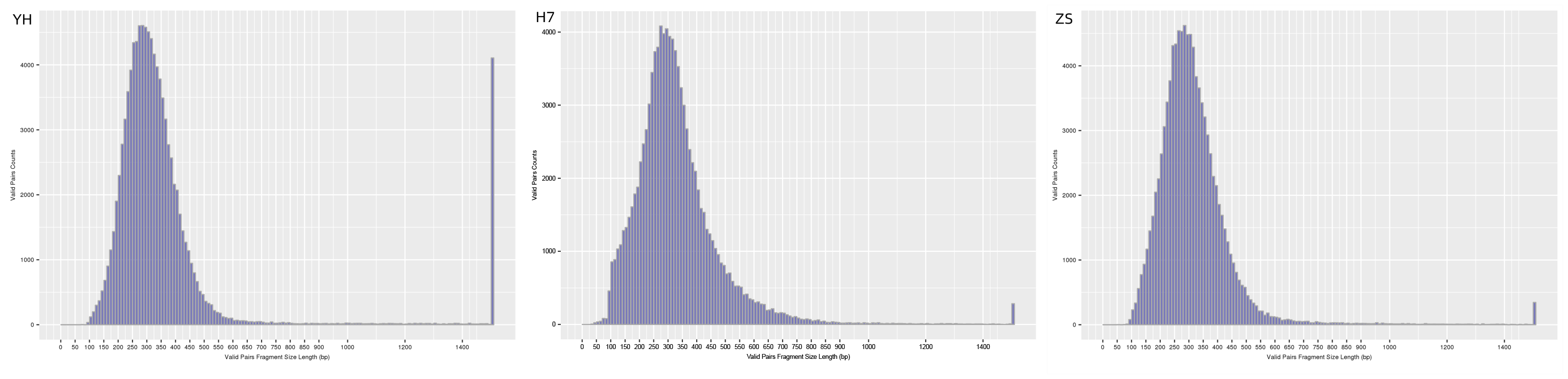

Supplement: Supplementary file 5 [file Image_2.PNG]

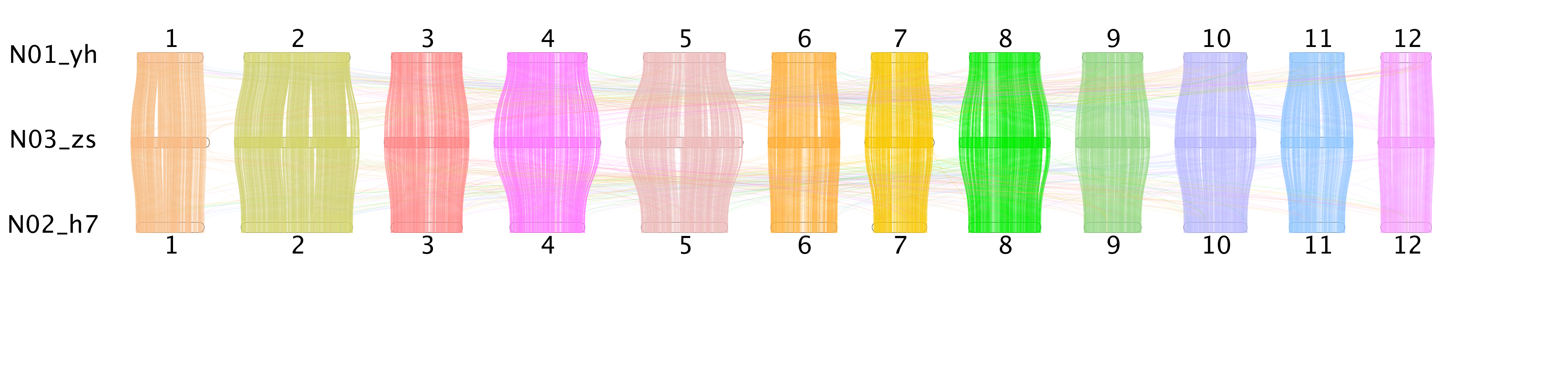

Supplement: Supplementary file 6 [file Image_3.PNG]
